# Analysis of spatial and temporal impact differences of birth rate in mainland China

**DOI:** 10.1038/s41598-022-22403-w

**Published:** 2022-10-17

**Authors:** Jinwei Zhang, Shuzhen Ding, Xijian Hu

**Affiliations:** College of Mathematics and System Science, Xinjiang University, Urumqi, 830046 Xinjiang China

**Keywords:** Environmental impact, Statistics, Anthropology

## Abstract

The continuing decline in the birth rate has led to a series of problems, such as the disproportion of population structure and severe aging population, which have restricted the country’s economic development. To have a deeper understanding of the geographical differences and influencing factors of the birth rate, this paper collects and organizes the birth population data of 31 provinces in mainland China from 2011 to 2019. The national region is divided into seven natural geographical regions to obtain the spatial hierarchy, and a hierarchical Bayesian Spatio-temporal model is established. The INLA algorithm estimates the model parameters. The results show significant spatial and temporal differences in birth rates in mainland China, which are reflected mainly in the combination of spatial, temporal, and Spatio-temporal interaction effects. In the spatial dimension, the northeast is low, the northwest and southwest are high, and the birth rate has an upward trend from east to west. These trends are caused by unbalanced economic development, different fertility attitudes and differences in fertility security, reflecting regional differences in spatial effects. From 2011 to 2019, China’s birth rate showed an overall downward trend in the time dimension. However, all regions except the northeast saw a significant but temporary increase in birth rates in 2016 and 2017, reflecting the temporal effect difference in birth rates.

## Introduction

The population is the foundation of sustainable development. When an economy grows to a certain magnitude, population aging is inevitable due to declining fertility, increasing life expectancy, and population migration^1,2^. The world’s major economies are facing the problem of population aging, and the global fertility decline has become an inevitable trend^3–5^. As the world’s largest developing country, although China’s total population is still rising, the birth rate has declined, and the problem of population aging is becoming more and more severe^6,7^. China has made significant adjustments to population policy to supply the population with more suitable for the demand of steady economic growth. For example, China began implementing the ”universal two-child ” policy in January 2016, which allowed all families to have two children^8,9^. Actually, China’s birth rate increased in 2016 and 2017, then reached 13.57% and 12.64% respectively, but then entered a period of decline. In the long run, the continued decline in the birth rate will bring a series of negative impacts, such as the imbalance of the population structure, the increased retirement burden of the working population, the deepening of social conflicts, and the economy development will be restricted in the future and so on. Therefore, the birth rate problem has become a hot topic in the current society and a critical problem that the Chinese government urgently needs to solve.

The problem of regional differences has always been one of the most common and essential problems all around the world^10–12^. With the increasingly significant regional differences in population, scholars from various countries began to analyze population data from a spatial perspective. For example, Velarde et al.^5^ analyzed the fertility trend and influencing factors of young women in Chile from 1992 to 2012. Their study found that the average fertility rate of adolescents has declined by 25% over the past 20 years, the affluent areas are lower than the poor areas, and fertility rates varied considerably between regions. Nandi et al.^4^ analyzed the trends and influencing factors of the total birth rate and repeat birth rate among Georgia adolescents from 2008 to 2016. Their study found that adolescents’ overall fertility and repeated fertility rates have decreased significantly since 2008, especially in areas with poor reproductive health care conditions. With the deepening of China’s reform and opening up, the regional development differences problem has become increasingly prominent, which has become an essential factor affecting social harmony^13–16^. Hu^17^ described the uneven geographical distribution of China’s population and the economy as early as the 1930s. He divided China into two parts of similar size, east and west, with the western part accounting for 3.7% of the country’s total population. In contrast, the eastern part accounted for 96.3% of the country’s total population. Zhang et al.^3^ used descriptive statistics and binary logistic regression analysis methods to analyze the fertility willingness and influencing factors of the population in mainland China. Their study found that fertility intentions will inevitably decline due to the lack of a good fertility environment, rising education levels and increasing monthly household income. Wu et al.^18^ used a spatial econometric model to analyze the spatial pattern characteristics and driving factors of population aging in China. Their study found that uneven economic development is the main reason for China’s aging population, which is high in the east and low in the west. Wei et al.^19^ analyzed the distribution characteristics and dynamic laws of the Chinese population using the spline-based method. Their study found that the economic development of China’s provinces is uneven, the growth rate of the resident population varies significantly, the total population growth rate is declining rapidly, and the problem of population aging is prominent. Chen et al.^20^ analyzed the regional differences and influencing factors of population aging in China from 1995 to 2011 using the Theil index method. Their study found that the regional differences in population aging in China are apparent, fluctuating repeatedly and increasing gradually.

At present, China’s population structure is undergoing significant changes. With the birth rate becoming the focus of sociology, the regional difference in population birth rate has become a new hotspot in geographical research. However, studies that analyze the population structure at multiple spatial levels are still rare, and our research has just made up for this gap. In this article, the spatial regions are stratified by natural geographic regions, and a hierarchical Bayesian Spatio-temporal model is established. The model parameters are estimated by the integrated nested Laplace approximations^21–23^ (INLA) algorithm . This study reveals the regional differences and temporal development trend of birth rate by analyzing the birth population and influencing factors in the Chinese Mainland. In addition, this study can not only serve as a reference for studying regional differences in birth rate but also put forward relevant suggestions and practical measures.

## Results

**Figure 1:**
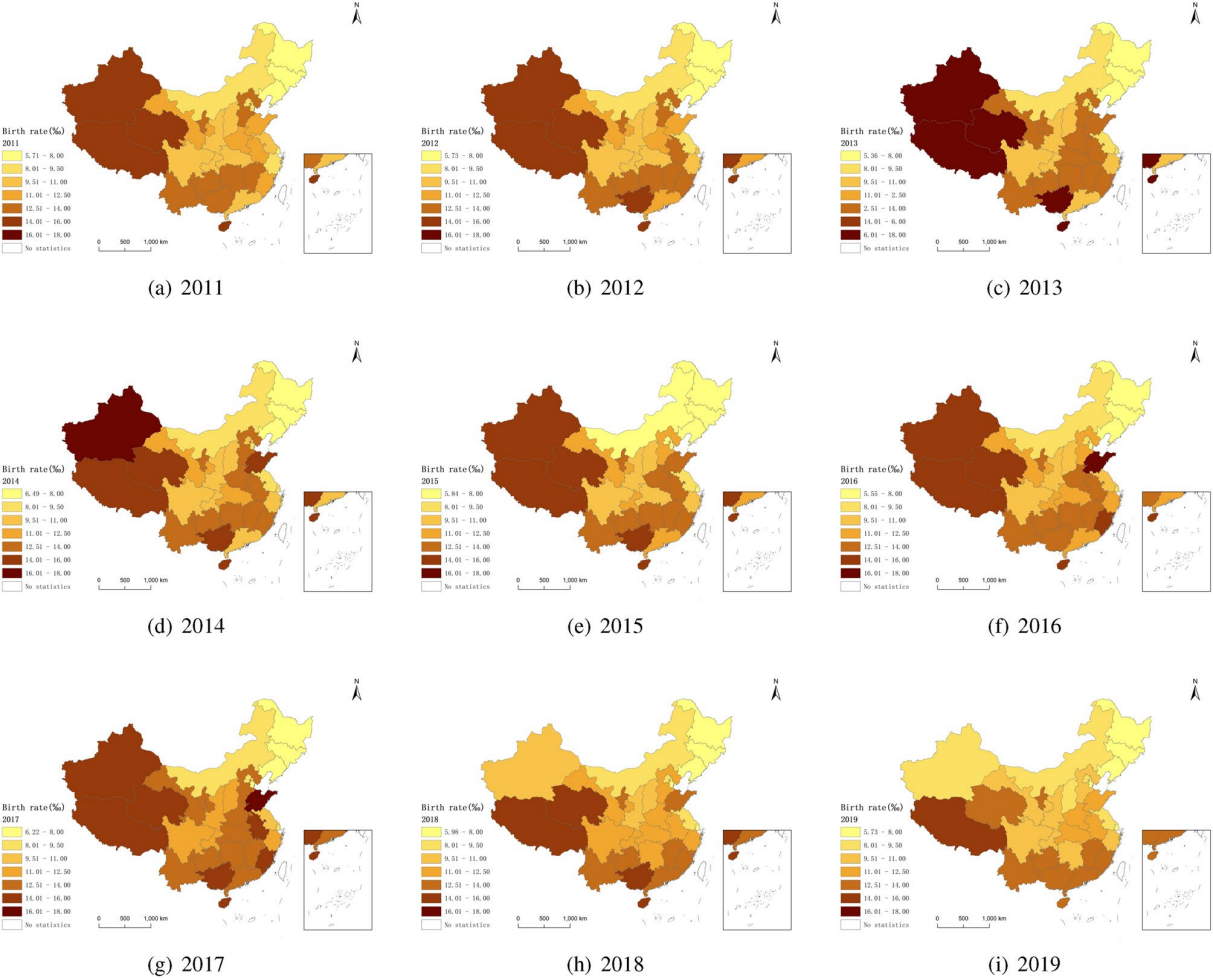
The trend of birth rate changes in 31 provinces in mainland China from 2011 to 2019.

From 2011 to 2019, the birth rate of 31 provinces in mainland China is shown in Fig. 1. In the spatial dimension, the birth rate in northeast China is low, while that in northwest and southwest China is high, and shows a trend of gradually increasing from east to west, with obvious spatial heterogeneity. From 2011 to 2013, the birth rate in northwest, southwest and central China showed an upward trend, and from 2014 to 2015, it showed a downward trend. From 2016 to 2017, the birth rate in most parts of China, except northeast China, showed an apparent upward trend. It declined gradually after 2018, especially in northwest, southwest, central and South China.

### Geographical differences

The geographic variation of birth rates in mainland China is mainly reflected in two levels, level 1 and level 2. The spatial effect on the birth rate is influenced by the spatial effect on two levels, that is, under the influence of the spatial effect on the level 2, it is affected by the spatial effect on the level 1. where the relative risk of spatial effects on level 1 $exp\{\mu _{i} + v_{i}\}$, the relative risk of spatial effects on level 2 $exp\{ \omega _{j} + \xi _{j } \}$, and the common relative risk of spatial effects on both levels $exp\{\mu _{i} + v_{i} + \omega _{j } + \xi _{j } \}$ are shown in Tables 1, 2, and 3, respectively. Under the combined effect of the two levels of spatial effects, Guangdong have the lowest relative risk and less than 0.5, and Beijing, Hainan, Zhejiang, Guangxi,Ningxia, Hebei, Hunan, Tibet, Tianjin, Guizhou,Shaanxi, Jiangxi, Qinghai, Xinjiang and Henan have higher relative risk. The results show that the spatial effect of Guangdong, Inner Mongolia, Sichuan, Chongqing, Shandong, Jiangsu, Shanxi, Hubei, Fujian, Shanghai, Jilin, Yunnan, Anhui, Liaoning, Heilongjiang and Gansu has a negative effect on the birth rate, while the spatial effect of Beijing, Hainan, Zhejiang, Guangxi, Ningxia, Hebei, Hunan, Tibet, Tianjin, Guizhou,Shaanxi, Jiangxi, Qinghai, Xinjiang and Henan has a higher positive effect on the birth rate.

**Table 1:** Relative risk distribution of spatial effects on level 1 .

Risk level	Relative risk interval	Province
Low	(0, 0.5]	Guangdong
Low	(0.5, 1.0]	Inner Mongolia, Chongqing, Sichuan, Shanxi, Guangxi, Beijing, Ningxia, Tibet, Hainan ,Tianjin ,Guizhou, Hebei, Yunnan
High	(1.0, 1.5]	Gansu, Xinjiang, Shaanxi, Jiangxi, Qinghai, Shandong, Jiangsu,Jilin, Hubei,Fujian, Shanghai, Anhui, Zhejiang, Liaoning, Henan, Heilongjiang , Hunan

**Table 2:** Relative risk distribution of spatial effects on level 2.

Risk level	Relative risk interval	Natural geographical regions
Low	(0, 0.5]	None
Middle	(0.5, 1.0]	Northeast China, Northwest China, North China, Southwest China
High	(1.0, 1.5]	Central China , South China, Eastern China

**Table 3:** Relative risk distribution for the combination of spatial effects on both levels.

Risk level	Relative risk interval	Province
Low	(0, 0.5]	Guangdong
Middle	(0.5, 1.0]	Inner Mongolia, Sichuan, Chongqing, Shandong, Jiangsu, Shanxi, Hubei, Fujian, Shanghai, Jilin, Yunnan, Anhui, Liaoning, Heilongjiang, Gansu
High	(1.0, 1.5]	Beijing, Hainan, Zhejiang, Guangxi,Ningxia, Hebei, Hunan, Tibet, Tianjin, Guizhou,Shaanxi, Jiangxi, Qinghai, Xinjiang, Henan

### Time differences

**Figure 2:**
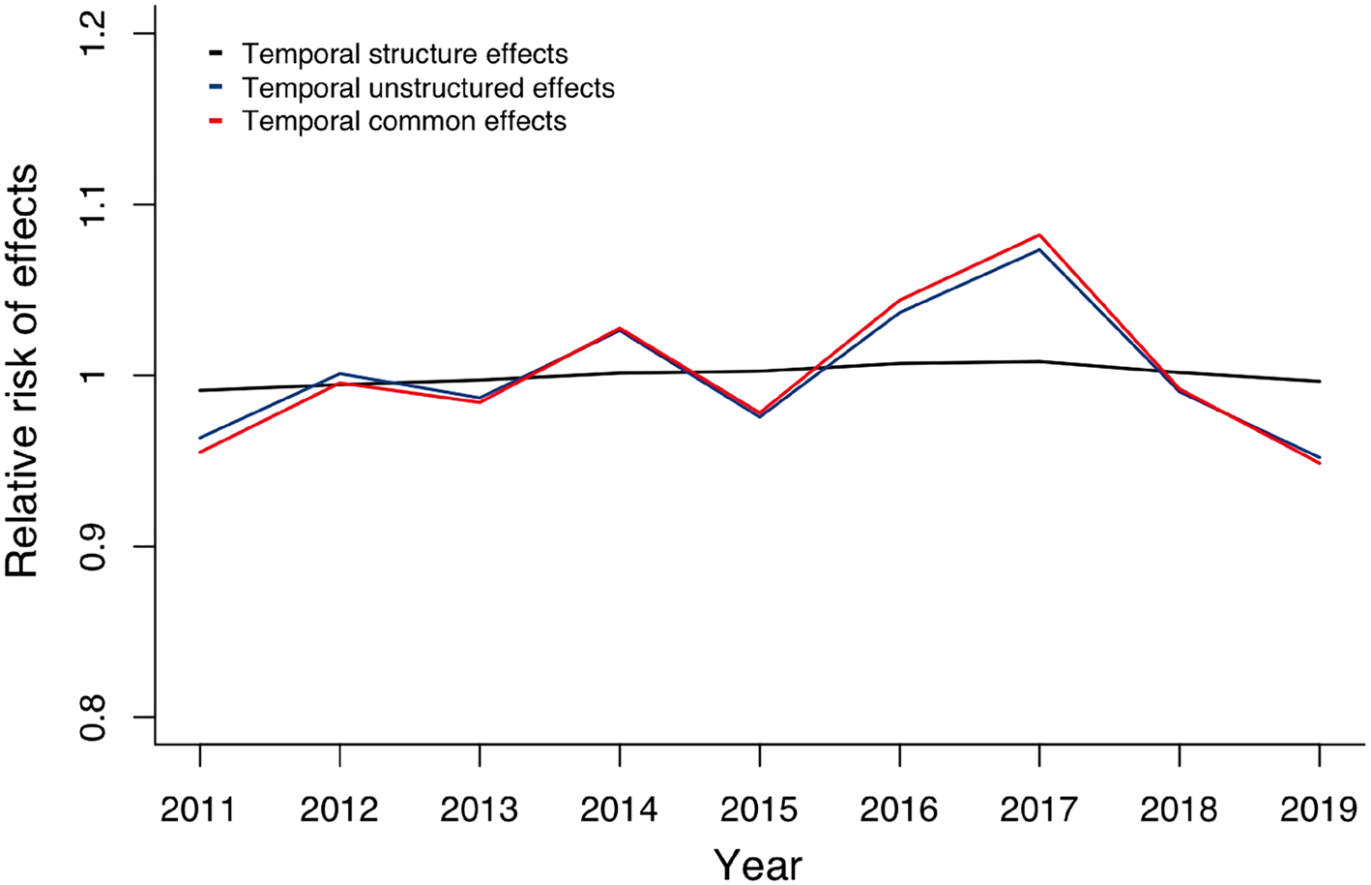
The posterior mean of the relative risk of the temporal effects in the model (1).

During the period 2011 to 2019, the birth rate in mainland China is subject to a combination of temporal structural and temporal unstructured effects. The relative risk of temporal structure effect is $exp\{\gamma _{t } \}$, the relative risk of temporal unstructured effect is $exp\{\phi _{t} \}$, and the relative risk of temporal combined effect is $exp\{\gamma _{t} + \phi _{t} \}$ is shown in Fig. 2. From 2011 to 2019, the impact of temporal unstructured effect $\phi _{t}$ was almost unchanged, and the relative risk level remained at about 1. From 2011 to 2015, the risk level of temporal structure effect $\gamma _{t }$ fluctuated around 1. After an obvious upward trend from 2015 to 2017, it began to decline from 2018. From the perspective of the overall time effect, from 2011 to 2015, the combined effect of the temporal structured effect $\gamma _{t }$ and temporal unstructured effect $\phi _{t}$ on fertility fluctuated between negative and positive. From 2015 to 2017, the time common effect positively impacted the fertility rate, and it increased yearly. After 2017, the positive impact of time common effect on fertility gradually decreased and finally became negative.

### Spatio-temporal differences

**Figure 3:**
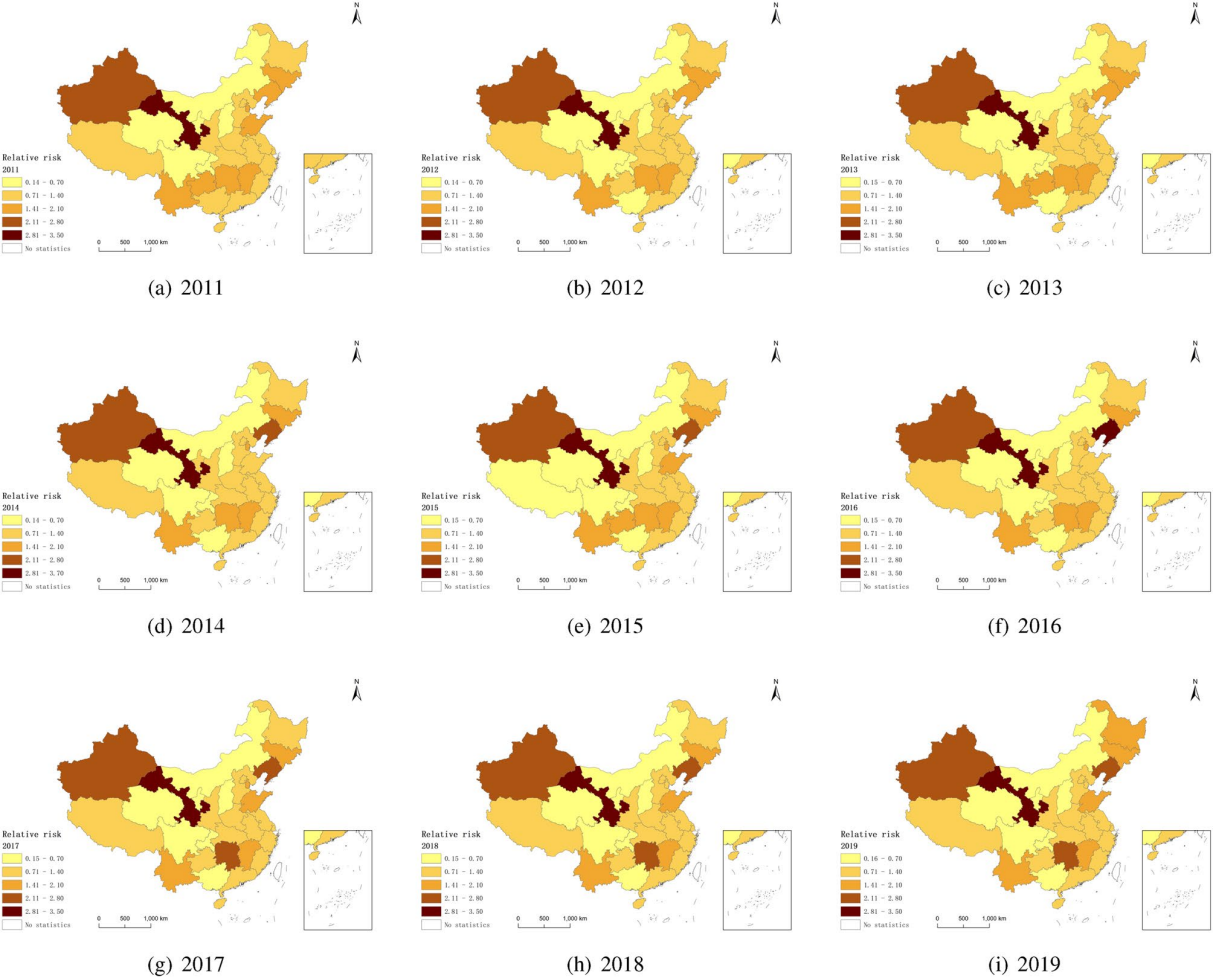
The relative risk under the combined effects of spatio-temporal interaction on two levels.

Figure 3 shows the trend chart of the relative risk $exp \{ \delta _{it }+\zeta _ {jt } \}$ of the common space-time interaction effect at the two levels between 2011 and 2019. In this study, the space-time interaction effect is the residual explanation of fertility under the space effect and time effect. As a substitute for the unknown unobservable effect, it is used to explain the deviation trend of space and time and depict the potential spatial pattern that fluctuates with time. The influence of spatial pattern on level 1 will show structural change with time trend, reflecting the interaction effect of spatial structural effect and time structure effect. The influence of spatial pattern on level 2 will not show structural spatial change with time trend, reflecting the interaction effect of spatial random effect and time random effect. Under the combined effect of Spatio-temporal interaction effects at the two levels, The main manifestations of Spatio-temporal trends are as follows: From 2011 to 2019, the relative risk of the Spatio-temporal interaction effect did not show a clear change trend. Among them, the Spatio-temporal interaction effects of Gansu, Liaoning, Hunan, and Xinjiang significantly positively impact the birth rate. In contrast, the Spatio-temporal interaction effects of Inner Mongolia, Shanxi, Qinghai, Sichuan, Chongqing, and Guangxi have a relatively high negative impact on the birth rate.

## Conclusion

In this paper, we establish a hierarchical Bayesian Spatio-temporal model and estimate the model parameters by the INLA algorithm, to explore geographic differences and temporal trends of the birth rates in mainland China from 2011 to 2019. The results show that the economic development is unbalanced, the birth rate is generally declining, the population growth rate is slowing down, and the population aging is severe in the Chinese Mainland. The birth rate has apparent spatial heterogeneity, and the difference between provincial regions is more significant than in natural geographical regions. The spatial dimension mainly shows that the northeast is low, the northwest and southwest are high, and the birth rate has an upward trend from east to west. These trends are caused by unbalanced economic development, different fertility attitudes, and differences in fertility security, reflecting regional differences in spatial effects. Regarding the time dimension, the changing birth rate trend is mainly affected by the overall economy and fertility policy that year, reflecting the temporal effect difference in birth rates. From 2011 to 2019, the birth rate mainly showed a downward trend. Although in 2016 and 2017, the birth rate in most parts of China established a significant increase, the rise gradually declined after 2018. For example, the birth rate growth from 2015 to 2017 is related to the “universal two-child” policy in 2016. However, due to the lack of a relevant security system after the universal two-child policy, residents’ growth level of per capita disposable income could not meet the burdens caused by the second child’s birth, so the birth rate began to decline again after 2017. It shows that the country can temporarily affect its birth rate through policies like allowing all families to have two children. However, to achieve stable growth in the birth rate, it is still necessary to establish full fertility protection measures in the follow-up.

Based on the results of this study, this paper makes the following recommendations: first, it is necessary to speed up the process of economic construction in the western region, further reduce the problem of unbalanced and insufficient development among various areas in mainland China, and promote long-term balanced population development. Second, we must raise the fertility awareness of all residents, especially in economically developed areas such as North China and South China. Residents’GDP and average disposable income are high, but the birth rate is low, and the birth rate has excellent room for improvement. Third, we must protect women’s legitimate rights and interests, establish a comprehensive assistance and security system for families with two or three children, and further implement maternity security and supporting measures for residents in various regions.

## Methods

### Data source

Considering data acquisition’s feasibility and completeness, this paper selects the birth population data of 31 provinces in mainland China from 2011 to 2019. To fully reflect the trend of geographical differences and try to minimize the interpretation of other influencing factors, this article interprets the influencing factors in the space as a spatial effect, analyzes the influencing factors in time as the time effect, and interprets the remaining effects as an inseparable Spatio-temporal interaction effect. Since the seven natural geographical divisions are based on science and follow the relevant zoning principles, the seven natural geographical divisions are regarded as large-scale, and the provincial regions are considered small-scale spatial stratification. Because the detailed data of the east, central and west of Inner Mongolia and the south-central and northeast of Hebei are not available, those are divided into north China in this study. The seven natural geographical regions are coded as $j = 1,2,\ldots ,7$, the province are coded as $i = 1,2,\ldots , 31$, and the time are coded as $t = 1,2,\ldots , 9$. Finally, summarize the data as a physical geography spatiotemporal data of districts, provinces, and time classification. The above data on births are taken from the National Bureau of Statistics (http://www.stats.gov.cn/tjsj/).

### Modeling

Construction of a two-level Poisson spatial multiscale model based on hierarchical Bayesian Spatio-temporal model^24^:

1 $$\begin{aligned} \left\{ \begin{array}{l} Y_{it } \sim {\text {Poisson}} ( \lambda _{it}) \\ \lambda _{it } = E_{it } \rho _{it } ~\\ \log ( \rho _{i t }) = \alpha + \mu _{i }+v_{i}+\gamma _{t }+\phi _{t}+\delta _{i t }+{\omega _{j }}+{\xi _{j }}+{\zeta _{jt } } , \end{array}\right. \end{aligned}$$

where $i=1, \ldots , 31, j=1, \ldots , 7$, and $i \in j$. *i* and *j* represent the observation indices at the provincial and natural geographical regions scales, respectively. $i \in j$ represents the smaller provincial scale nested in the larger Natural geographical regions scale. The provincial and the Natural geographical regions scale are called level 1 and level 2, respectively. *t* represents the time observation index, $t=1, \ldots , 9$. $Y_{it}$ represents the number of births on the *i*th region in year *t*. $E_{it}$ represents the expected number of births in the *i*th region in year *t*. $\rho _{it}$ represents the relative risk of births. $\alpha$ stands for the average log relative risk. $\mu _{i}$ and $v_{i}$ represent the spatial structural and unstructured spatial effects at level 1, respectively, forming the spatial effect at level 1. $\gamma _{t}$ and $\phi _{t}$ represent the time structural and unstructured time effects at level 1, and their sums together form the time effect. ${\omega _{j }}$ and ${\xi _{j }}$ represent the structural and unstructured spatial effects at level 2, respectively, which together form the spatial effect at level 2. $\delta _{it}$ and $\zeta _{jt}$ are used as spatio-temporal interaction effects on level 1 and level 2, respectively, and the four types of Spatio-temporal interactions^25^ are shown in Table 4.

**Table 4:** Four types of spatio-temporal interaction.

Interaction type	Spatio-temporal interaction on level 1		Spatio-temporal interaction on level 2	
	Parameter interaction	Structural matrix	Parameter interaction	Structural matrix
Type 1	$v_{i} \otimes \phi _{t}$	$R_{v_{i}} \otimes R_{\phi _{t}}$	$\xi _{j} \otimes \phi _{t}$	$R_{\xi _{j}} \otimes R_{\phi _{t}}$
Type 2	$v_{i} \otimes \gamma _{t}$	$R_{v_{i}} \otimes R_{\gamma _{t}}$	$\xi _{j} \otimes \gamma _{t}$	$R_{\xi _{j}} \otimes R_{\gamma _{t}}$
Type 3	$\phi _{t} \otimes \mu _{i}$	$R_{\phi _{t}} \otimes R_{\mu _{i}}$	$\phi _{t} \otimes \omega _{j}$	$R_{\phi _{t}} \otimes R_{\omega _{j}}$
Type 4	$\mu _{i} \otimes \gamma _{t}$	$R_{\mu _{i}} \otimes R_{\gamma _{t}}$	$\omega _{j} \otimes \gamma _{t}$	$R_{\omega _{j}} \otimes R_{\gamma _{t}}$

### Priority specification

The INLA algorithm is a fast calculation method proposed by Rue et al.^21^ for latent Gaussian models that satisfy the Gaussian Markov random field conditions. In the INLA algorithm, the latent random field of model (1) is $\varvec{x} = \{ \varvec{\eta }, \alpha , \varvec{\mu } , \varvec{v} , \varvec{\gamma } , \varvec{\phi } ,\varvec{\delta } , \varvec{\omega } , \varvec{\epsilon } , \varvec{\zeta } \}$, the set of hyperparameters is $\varvec{\theta }=\{ \tau _{\mu }, \tau _{v} , \tau _{\gamma }, \tau _{\phi },\tau _{\delta }, \tau _{\omega }, \tau _{\xi }, \tau _{\zeta } \}$, the distribution of hyperparameters is $log\varvec{\theta } \sim log ~Gamma (0.5, 0.000 5)$, with precision matrix $Q(\varvec{\theta })$. The prior information on the spatial structure effects $\{ \mu _{i}, \omega _{j} \}$ is expressed by the intrinsic conditional autoregressive model^25–27^ (ICAR). Let $m = \{ \mu _{i}, \omega _{j} \}$, then its conditional distribution is:

$$\begin{aligned} m_{c} \mid m_{d}, c \ne d, \tau _{m} \sim {\text {Normal}}\left( \frac{1}{n_{c}} \sum _{d=1}^{n} a_{cd}m_{d}, \frac{1}{n_{c} \tau _{m}}\right) , \end{aligned}$$

where $\tau _{m}=\left\{ \tau _{\mu }, \tau _{\omega }\right\} , \tau _{\mu }$ and $\tau _{\omega }$ are the accuracy parameters of the spatial structure effects on level 1 and level 2, respectively. *n* represents the number of regions, $n=31$ when modeling $\mu _{i}$ and $n=7$ when modeling $\omega _{j}$. $c \sim d$ represents the region *c* adjacent to the region *d*. $n_{c}$ represents the number of neighboring regions that share an edge with region *c*. The spatial correlation between regions is defined by their spatial adjacency:

$$\begin{aligned} a_{c d}=\left\{ \begin{array}{l} 1 \qquad ~ c \sim d \\ 0 \qquad \text{ others } . \end{array}\right. \end{aligned}$$

The temporal structure effect $\gamma _{ t}$ obeys the first-order random walk model^25,26^ (RW1), whose conditional distribution is:

$$\begin{aligned} \gamma _{t} \mid \gamma _{t-1} \sim {\text {Normal}} (\gamma _{t-1}, \sigma _{\gamma }^{2} ) , \end{aligned}$$

where $\sigma _{\gamma }^{2}=\frac{1}{\tau _{\gamma }}$, $\tau _{\gamma }$ is the precision parameter for the time structure effect. The fixed effect is denoted $s = \{\alpha \}$, and its prior information is modeled as $s \sim N(0,10^{6})$. The unstructured effect is denoted $g = \{v_{i}, \xi _{j}, \phi _{t}\}$, whose prior information is modeled as $g \sim Normal(0,\frac{1}{\tau _{g} } )$. Where $\tau _{g} = \{ \tau _{v},\tau _{\xi },\tau _{\phi } \}$, $\tau _{v}$, $\tau _{\xi }$ and $\tau _{\phi }$ represent the accuracy parameters of the unstructured spatial effect at level 1, the unstructured spatial effect at level 2, and the unstructured time effect, respectively. The Spatio-temporal interaction effect is denoted as $r = \{ \delta _{i t }, {\zeta _{jt}} \}$, and its prior information is modeled as $r \sim Normal(0,\frac{1}{\tau _{r} R_{r}} )$. where $\tau _{r} = \{ \tau _{\delta }, \tau _{\zeta } \}$ is an unknown scalar, and $R_{r} = \{ R_{\delta }, R_{\zeta } \}$ is a structure matrix that denoted as the Kronecker product^25^, set by the interaction type in Table 4.

### Model selection

Model (1) has four Spatio-temporal interaction types at level 1 and level 2, respectively. However, due to the superposition of interaction types at two levels, model (1) has 16 choices in Spatio-temporal interaction. In order to find the most appropriate interaction type for model (1) at both levels, we first disregard the spatial and Spatio-temporal interaction effects at level 2. At this point, we can get the model (2):

2 $$\begin{aligned} \left\{ \begin{array}{l} Y_{it } \sim {\text {Poisson}} ( \lambda _{it}) \\ \lambda _{it } = E_{it } \rho _{it }~\\ \log ( \rho _{it }) = \alpha + \mu _{i }+v_{i}+\gamma _{t }+\phi _{t}+\delta _{i t } . \end{array}\right. \end{aligned}$$

The selection information of four spatiotemporal interaction types on level 1 of the model (2) is shown in Table 5. DIC represents the deviance information criterion^28,29^, a statistical indicator to compare Bayesian models’ fitting effect and complexity. WAIC represents the widely applicable informationcite^30,31^, which is not affected by parameterization and is close to the Bayesian cross-validation results. It can be concluded from Table 5 that the DIC and WAIC of the model (2) under type 2 are the smallest, so chosen type 2 as the Spatio-temporal interaction type on level 1 of the model (1). After determining the Spatio-temporal interaction type on level 1 of the model (1), the DIC and WAIC under the four Spatio-temporal interaction types on level 2 are shown in Table 6. By comparing the values of DIC and WAIC in Table 6, type 3 is selected as the Spatio-temporal interaction type on level 2 of the model (1).

**Table 5:** The selection information for the four spatio-temporal interaction types on level 1 of the model (2).

Interaction type	Structural matrix	DIC	WAIC
Type 1	$R_{v_{i}} \otimes R_{\phi _{t}}$	3353.325	3278.675
Type 2	$R_{v_{i}} \otimes R_{\gamma _{t}}$	3339.704	3286.121
Type 3	$R_{\phi _{t}} \otimes R_{\mu _{i}}$	3349.765	3275.086
Type 4	$R_{\mu _{i}} \otimes R_{\gamma _{t}}$	3329.670	3273.612

**Table 6:** The selection information for the four spatio-temporal interaction types on level 2 of the model (1).

Interaction type	Structural matrix	DIC	WAIC
Type 1	$R_{\xi _{j}} \otimes R_{\phi _{t}}$	3327.876	3270.331
Type 2	$R_{\xi _{j}} \otimes R_{\gamma _{t}}$	3329.660	3271.466
Type 3	$R_{\phi _{t}} \otimes R_{\omega _{j}}$	3330.109	3272.774
Type 4	$R_{\omega _{j}} \otimes R_{\gamma _{t}}$	3329.675	3271.025

## Data Availability

All data generated during the current study are available in the National Bureau of Statistics (http://www.stats.gov.cn/tjsj/). China Electronic Maps comes from the Standard Map Service Website of the Ministry of Natural Resources (https://www.tianditu.gov.cn/), and all maps are based on the standard map with the drawing review number of GS (2019) 1822, and the base map is not modified. The software used in this paper is R: A Language and Environment for Statistical Computing, version 4.2.1 (https://www.R-project.org/).
